# Optimization of Serine Protease Purification from Mango (*Mangifera indica* cv. Chokanan) Peel in Polyethylene Glycol/Dextran Aqueous Two Phase System

**DOI:** 10.3390/ijms13033636

**Published:** 2012-03-19

**Authors:** Amid Mehrnoush, Shuhaimi Mustafa, Md. Zaidul Islam Sarker, Abdul Manap Mohd Yazid

**Affiliations:** 1 Department of Food Technology, Faculty of Food Science and Technology, Universiti Putra Malaysia, 43400 UPM Serdang, Selangor, Malaysia; E-Mail: Mehrnoush_amid@yahoo.com; 2 Department of Microbiology, Faculty of Biotechnology and Biomolecular Science, Universiti Putra Malaysia, 43400 UPM Serdang, Selangor, Malaysia; E-Mail: shuhaimi@biotech.upm.edu.my; 3 Department of Pharmaceutical Technology, Faculty of Pharmacy, International Islamic University Malaysia, Kuantan Campus, Bandar Indera Mahkota, 25200 Kuantan, Pahang, Malaysia; E-Mail: zaidul@iium.edu.my

**Keywords:** purification, polyethylene glycol (PEG), serine protease, mango peel, yield

## Abstract

Mango peel is a good source of protease but remains an industrial waste. This study focuses on the optimization of polyethylene glycol (PEG)/dextran-based aqueous two-phase system (ATPS) to purify serine protease from mango peel. The activity of serine protease in different phase systems was studied and then the possible relationship between the purification variables, namely polyethylene glycol molecular weight (PEG, 4000–12,000 g·mol^−1^), tie line length (−3.42–35.27%), NaCl (−2.5–11.5%) and pH (4.5–10.5) on the enzymatic properties of purified enzyme was investigated. The most significant effect of PEG was on the efficiency of serine protease purification. Also, there was a significant increase in the partition coefficient with the addition of 4.5% of NaCl to the system. This could be due to the high hydrophobicity of serine protease compared to protein contaminates. The optimum conditions to achieve high partition coefficient (84.2) purification factor (14.37) and yield (97.3%) of serine protease were obtained in the presence of 8000 g·mol^−1^ of PEG, 17.2% of tie line length and 4.5% of NaCl at pH 7.5. The enzymatic properties of purified serine protease using PEG/dextran ATPS showed that the enzyme could be purified at a high purification factor and yield with easy scale-up and fast processing.

## 1. Introduction

Proteolytic enzyme (EC 3.4) is a class of proteins found in various sources, including animals, plants and microorganisms because of its ubiquitous nature [1]. Proteases from plants show wide substrate specificity while at the same time have a broad range of temperature, pH also in the presence of additives and other organic compounds [2]. Thus, this enzyme is widely used in many industrial processes such as food, detergents, waste, pharmaceutical and leather [3].

Mango is universally one of the most important commercial tropical fruits [4]. An FAO (Food and Agriculture Organization) report states that mango production accounts for an estimated 38% of total tropical fruit output, and in the processing of this fruit, peel and kernel are two important by-products [5]. Mango peel is currently unutilized for any commercial purposes and disposed of as waste, thereby becoming a source of pollution [6]. However, it has been discovered that there is commercial potential in mango peel as it can be a rich and cost effective source of natural enzymes [5].

High purity of serine protease is required in order to meet the demands of the wide range of industrial and scientific uses. The traditional purification processes are multistep, discontinuous, time and labor consuming, which all add to the cost and also could cause loss of product yield [7]. Today, industry demands fast and economic downstream processes for the purification of protein products, including those processes that give high yield and high purity of the product [8]. The aqueous two-phase system (ATPS) is one of the purification methods that can fulfill these criteria. This is because the operation environment is mild, scale-up is easy, process is rapid, cost of material is low and denaturation of the proteins is minimized [9,10].

The ATPS currently used is based on two polymers or one polymer and salt [11]. As an alternative, the polyethylene glycol (PEG)-salt ATPS has been used for large-scale enzyme processing. Although this system is inexpensive, the high salt concentration in the top and bottom phases limits its usefulness [12]. ATPS based on PEG-salt causes the denaturation of the biological structure at rather high ionic strength and also dissociation of the ligand-protein complex [13]. Waste disposal is another problem in employing the PEG-salt system. ATPS based on dextran has the advantage of being biodegradable [14]. Furthermore, dextran shows a stabilizing effect on enzymes, which apparently supports the high enzyme activity in the ATPS system [15,16]. Therefore, there is a need to improve the ATPS method at low concentrations of salt and it seems that the polymer-polymer system could be more useful than PEG-salt systems.

In our previously published paper [17], the procedure for extraction of serine protease from mango peel was indicated. At present, there is a lack of information about optimization of serine protease purification from mango (*Mangifera Indica* cv. Chokanan) peel by PEG/dextran ATPS. Thus in the present study the purification of the enzyme from mango peel using PEG/dextran will be described. The aim of this study is to investigate the effects of different parameters such as PEG molecular weight, TLL (Tie Line Length), NaCl and pH on serine protease partitioning, purification factor and yield in order to optimize the serine protease purification in PEG/dextran ATPS using response surface methodology (RSM).

## 2. Results and Discussions

### 2.1. Effect of PEG Molecular Weight on Partitioning of Serine Protease

**Figure 1:**
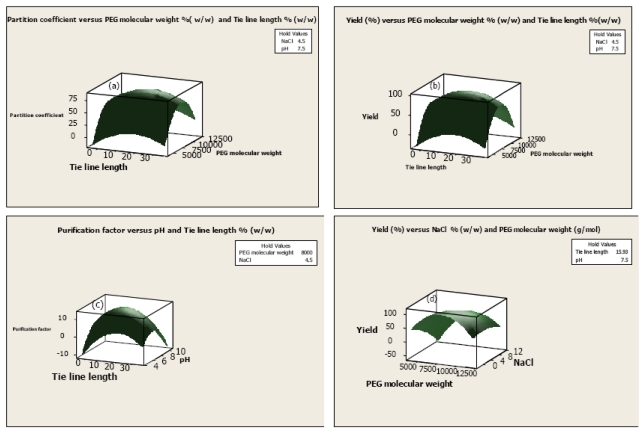
Response surface plots showing the interaction effects of (**a**) TLL and PEG molecular weight on partition coefficient; (**b**) TLL and PEG molecular weight on yield; (**c**) TLL and pH on purification factor; (**d**) PEG molecular mass and NaCl, on yield of serine protease.

Based on the results (Table 1), the main effect of PEG molecular weight was its high significant effect on partitioning of serine protease. It was observed that the maximum partition coefficient (84.2) was obtained at 8000 g·mol^−1^ of PEG molecular mass. Meanwhile, there was a decrease in partition coefficient at higher PEG molecular weight, because, in this condition, PEG gets more compact conformation with intermolecular hydrophobic bonds and prevents enzyme migration into the top phase, thus target enzymes should be transferred to the bottom phase. Also, purification factor and yield of serine protease were decreased at 10,000 g·mol^−1^ of PEG molecular weight. Reports have shown that when PEG chain length increases, available free volume to accommodate serine protease in the top phase decreases, resulting in a decrease of enzyme specific activity in this phase [18]. When a 6000 g·mol^−1^ of PEG molecular weight was used to purify the serine protease, the purification factor of enzyme also was decreased. This could be due to the decreased exclusion effect of PEG, and thus, both contaminate and target enzyme could be moved from bottom phase to the top phase and decrease the purity and yield of enzyme. Therefore, the intermediate molecular weight is the best choice for the ATPS purification of serine protease. As clearly shown in Figure 1a, the partition coefficient was significantly increased in the presence of 8000 g mol^−1^ of PEG molecular mass.

### 2.2. Effect of TLL on Serine Protease Partitioning

The partition coefficient and yield of serine protease were significantly influenced by the main and quadratic effects of TLL and the interaction effect of TLL and PEG molecular mass (Table 1). The partition coefficient and yield of serine protease were 18.3 and 34% at 6.25% (w·w^−1^) of TLL. It has been reported that increase in tie-line length causes to increment the hydrophobicity of the top phase as well as interfacial tension between two phases. Based on the results, 17.4% (w·w^−1^) TLL caused a better partitioning of serine protease into the top phase and further increase of the yield. The maximum partition coefficient and yield were 84.2 and 97.3% at TLL of 17.4% (w·w^−1^). Rito-Palomares and Hernández [19] reported that when TLL increases, the free volume of the bottom phase decreases, thus adding protein partitioning from bottom to the top phase or to the interface. Therefore, an increase in TLL caused an increase in the protein partition coefficient, resulting in an increase in the yield of serine protease.

At higher TLL [25.6% (w·w^−1^)], partition coefficient and yield were significantly decreased to 34 and 50.8%, respectively, which could possibly be due to reduction in the volume ratio of top phase thus serine protease activity and yield significantly decreased in this phase. Figure 1a,b indicates how significant (*p* < 0.05) interaction effects of TLL and PEG molecular mass influenced the partition coefficient and yield of serine protease. The figure shows that when TLL was increased from 0% (w·w^−1^) to 17.4% (w·w^−1^), partition coefficient and yield increased but the increase in TLL value from 17.4% (w·w^−1^) to 25.6% (w·w^−1^) led to a decrease in partition coefficient and yield of purified enzyme.

### 2.3. Effect of pH on Partitioning of Serine Protease

The main and quadratic effects of pH and interaction effect of pH and tie line length showed a highly significant (*p* < 0.05) effect on purification factor of serine protease (Table 1). An important parameter to control enzyme partitioning is pH. The purification factor of serine protease was significantly decreased at pH 6, perhaps because the denaturation of the enzyme was higher when the lower pH was applied. Also, at pH 6, with serine protease partitioned to the bottom phase, it showed that change of enzyme partition behavior was the effect of protein charge. When the pH of system changed, the partitioning of the serine protease was based on the net charge of the protein and surface properties other than the charge.

The isoelectric point (pI) of serine protease is about 6, thus the enzyme has negative charge above pH 7–9 and PEG tends to interact with negative charge. The results indicate that the highest purification factor (14.37) with improvement in partitioning of the enzyme was achieved at pH 7.5. According to the results, ATPS of pH 7.5 was chosen for the serine protease purification. Figure 1c shows the relationship between purification factor and interaction effect of independent variables.

### 2.4. Effect of NaCl on Partitioning of Serine Protease

**Figure 2:**
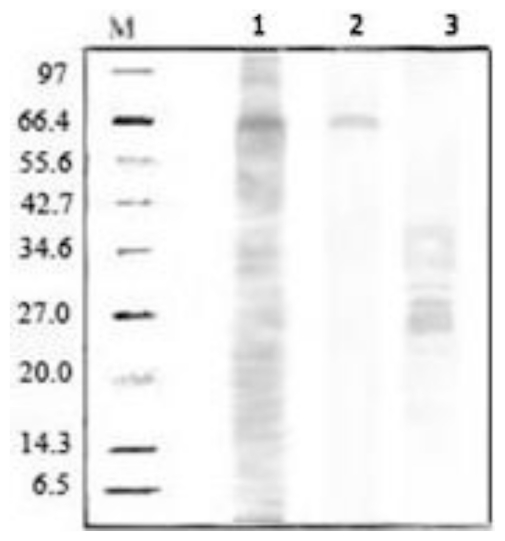
SDS-PAGE analyses on the serine protease, M = protein molecular markers (6.5–97 kDa); 1 = crude feedstock; 2 = ATPS top phase Lane; 3 = ATPS bottom phase.

The purification of the serine protease using the ATPS method showed that the main and quadratic effects of NaCl and the interaction effects of NaCl and PEG molecular mass indicated the significant (*p* < 0.05) effects on partitioning of the serine protease (Table 1). In general, the interaction of hydrophilic polymers and enzymes can be modified by addition of NaCl in ATPS [20]. The variation of NaCl concentration causes an electrical potential difference between two phases and results in enzyme partitioning. Based on the results, the highest yield (97.3%) of serine protease was obtained with the addition of 4.5% (w·w^−1^) of NaCl (Figure 1d). It showed that the hydrophobic interaction between PEG and hydrophobic surface of seine protease was significantly increased at this concentration of NaCl. However, the higher concentration of the salt had a negative effect on the partitioning of the enzyme because the unequal partitioning of natural salt between two phases affected the chemical potential of the solute. Figure 1d shows how the interaction effect of NaCl and PEG molecular mass significantly affects the yield of serine protease using ATPS. In addition, Figure 2 (Line 2) showed that the purity of purified serine protease after purification.

### 2.5. Validation of Empirical Equation

**Figure 3:**
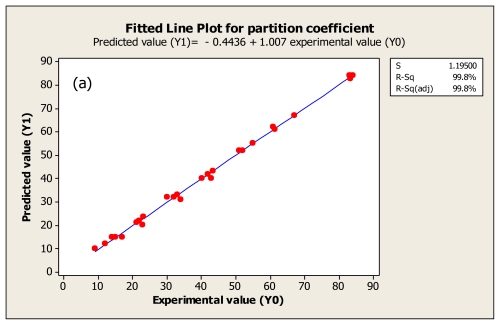

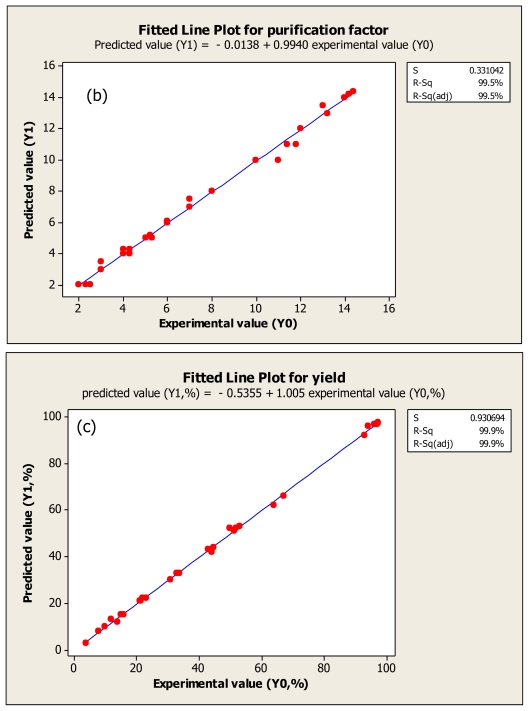
Fitted line plot indicating the closeness between predicted values (*Y*_1_) and experimental values (*Y*_0_) for serine protease partition coefficient (**a**), purification factor (**b**), yield (**c**).

Table 2 shows the estimated regression coefficient, *R*^2^ and adjusted *R*^2^ as long as *p*-value of regression for each response variable (partition coefficient, purification factor and yield) is given. As shown in the table, the *R*^2^ values are obtained for all response variables higher than 0.8 (0.993–0.997) and *p*-value of regression is less than 0.05. Therefore, according to a criterion for validation of model presented by Joglekar and May [21], the models are suitably and accurately employed for predicting the response variable as functional of independent variables. In addition, the comparison between experimental values and those predicted is shown in Figure 3a–c. It is seen there is overall closeness between experimental and predicted data, and based on Montgomery [22] the closeness between these variables indicates the adequacy of the regression equations.

## 3. Experimental Section

### 3.1. Materials

Mango fruits were purchased from local market (Selangor, Malaysia) in slightly under ripe commercial maturity stage with the brix of 14. All chemicals and reagents used were analytical grade. Dextran T500 (average molecular weight of 500,000 g·mol^−1^), polyethylene glycol (PEG) 4000, 6000, 8000, 10,000 and 12,000 (g·mol^−1^), bovine serum albumin (BSA), azocasein and Bicinchoninic acid solution were supplied by Sigma Chemical Co, (St. Louis, UK). Trichloroacetic acid (TCA), 99%, di-sodium hydrogen anhydrous and sodium hydrogen phosphate monohydrate were purchased from Merck (Darmstadt, Germany).

### 3.2. Extraction Procedure of Serine Protease from Mango Peel

Mango peel was obtained from 100 g fresh mangoes that had been washed with double distilled water. The peel was cut into small cubes (3 mm × 3 mm) and then blended with 50 mL sodium phosphate hydrogen at pH 7.5 by using a Waring commercial laboratory blender 32BL79 (Torrington, CT, USA) for 2 min at high speed at 4 °C. Crude enzyme extract was produced by putting the resultant homogenate through a cheesecloth filter and then centrifuging at 8000 g for 15 min at 4 °C [17].

### 3.3. Preparation of PEG/Dextran ATPS

Different phase systems were prepared for the selection of the suitable bottom phase and top phase [23] and then samples from each phase were taken and analyzed for the serine protease activity. A 50% (w·w^−1^) polymer by mass was prepared as the concentration of PEG stock solutions. This was followed by the preparation of phase systems in 15 mL graduated centrifuge tubes by weighing an appropriate amount of “dextran T-500” and PEG with different molecular weights (4000–12,000 g·mol^−1^), NaCl (−2.5–11.5% w·w^−1^), pH (4.5–10.5) and 20% (w·w^−1^) crude feedstock at room temperature. Then the appropriate amount of distilled water was added to the mixture to obtain a final mass of 10 g system. Thorough mixture of the content by gentle agitation for equilibration and then phase separation was achieved using centrifugation at 4000× g for 10 min. Then serine protease activity and protein concentration of top phase and bottom phase were determined [24,25].

### 3.4. Analytical Tests

#### 3.4.1. Proteolytic Activity Assays

Protease activity of purified enzyme was estimated according to the method described by Dosoretz *et al.* [26] with some modifications. The proteolytic reaction mixture contained 0.5 mL of enzyme solution and 0.5 mL of 0.2% (w·v^−1^) azo-casein prepared in 50 mM Tris-HCl (pH 8.0) buffer. The mixture was incubated in a water-bath at 70 °C for 1 h and 0.5 mL of (30% w·v^−1^) TCA was added to stop the reaction. The supernatant was obtained by centrifugation at 13400 rpm for 10 min (Microfuge 18 centrifuge, Beckman Coulter, Inc, Krefeld, Germany) and was then filtered through a 0.26 μm film. The absorbance at 410 nm was measured at spectrophotometer (BioMate™-3, Thermo Scientific, Alpha Numerix, Woodfield Dr, Webster, NY, USA). One unit of proteolytic activity is defined as the amount of enzyme causing an increase in absorbance of 0.01. The results are expressed as a mean of three readings with an estimated error of ±10%.

#### 3.4.2. Bicinchoninic Acid Assay

In order to determine the total protein concentration of the phase systems, the bicinchoninic acid method was adopted and bovine serum albumin (BSA) was used as the standard [27]. As described, 200 μL of the working reagent was added with an amount of 50 μL of the sample in a microtiter plate. This was then heated at 37 °C for half an hour. Absorbance was measured at 562 nm against a blank reagent.

#### 3.4.3. Determination of Partition Coefficient, Purification Factor and Yield

The partition coefficient (*K*) was determined by taking the ratio of serine protease activity in top phase (*A*_T_) and dividing it by enzyme activity in bottom phase (*A*_B_) (Equation 1).

(1) $$K=A_{\text{T}}/A_{\text{B}}$$

The specific activity of the enzyme was determined by taking the ratio of total activity of serine protease and dividing it by serine protease total protein (Equation 2):

(2) $$\text{Specific activity }(\text{U }{\text{mg}}^{-1})=\text{Total activity }(\text{U})/\text{Total protein}\;(\text{mg})$$

Based on Equation 3, the specific activity of serine protease in the top phase divided by the specific activity of the enzyme in the bottom phase gives the purification factor of serine protease in the top phase (*P*_FT_):

(3) $$P_{\text{FT}}=\text{Specific activity of top phase sample}/\text{Specific activity of crude feedstcok}$$

The yield of serine protease in the top phase (*Y*_T_) is determined using Equation 4:

(4) $$Y_{\text{T}}(\%)=100/1+(1/[V_{\text{R}}]\cdot [K_{\text{e}}])$$

where *K*_e_ is the partition coefficient of serine protease and *V*_R_ is the volume ratio of the top phase to the bottom phase [28].

### 3.5. Experimental Design and Statistical Analysis

RSM was used to determine the effects of four independent variables in ATPS, namely PEG molecular weight (4000–12,000 g·mol^−1^, *x*_1_), TLL (−3.42–35.27% (w·w^−1^), *x*_2_), NaCl (−2.5–11.5%, (w·w^−1^), *x*_3_) and pH (4.5–10.5, *x*_4_) on partition coefficient (*Y*_1_), purification factor (*Y*_2_) and yield (*Y*_3_) of the purified serine protease from mango peel. Central composite design (CCD) was the method employed in the assessment. The assessment of 30 supernatants on the basis of a CCD was done involving 16 factorial points, eight axial points (±α) and six center points (Table 3). There was a six-time repetition of the center point to determine the possibility of pure error. To determine the regression coefficient and statistical significance of the models, response surface analysis was carried out. An overall response surface model is as follows:

$$Y=\beta_{0}+\sum \beta_{i}x_{i}+\sum \beta_{ii}x_{i}^{2}+\sum \beta_{ij}x_{i}x_{j}$$

From the above equation, *Y* is response calculated by the model; *β*_0_ is a constant; *β_i_*, *β_ii_* and *β_ij_* are linear, squared and interaction coefficients, respectively. The estimated regression coefficients of the response surface models for the serine protease purification, along with the corresponding *R^2^* and adjusted *R^2^* values are presented in Table 2. The significant *p*-value of regression and *R^2^* with no less than 0.80 indicates a good model fit. As shown in the Table, the *p*-value of regressions is less than 0.05 and *R^2^* and adjusted *R^2^* values from 0.993 to 0.997 and 0.991 to 0.993 respectively, were obtained for all the response variables. Thus, the models of response surface were suitably and accurately used for predicting high variation percentage (≥80%) of the properties of purified serine protease as functional to the purification variables. Larger values of absolute t-value and smaller values of *p*-value indicate that the variables will be more significant (*p* < 0.05). Only the significant (*p* < 0.05) independent variable effects were included in the reduced model. The terms found to be statistically non-significant (*p* > 0.05) were removed and the experimental data were refitted to only the significant (*p* < 0.05) independent variable effects so as to achieve the final reduced model. It is worth noting that in a situation where some of the variables are not significant, they will still be kept in the final reduced model, as in the case of a quadratic or interaction term involving this significant (*p* < 0.05) variable [29,30]. The design matrix of the experiment, the analysis of the data and the optimizing procedure were all done using the Minitab v.14 statistical package (Minitab Inc., PA, USA).

### 3.6. Optimization and Validation Procedures

Numerical and graphical optimizations were performed to determine the optimum levels of independent variables. Numerical optimization was performed to determine the exact optimum level of independent variables, using a response surface optimizer (Minitab v.14). Graphical optimization (using three-dimensional response surface plot) can be employed to explain the response models and to be able to better comprehend the implications of interaction between independent variables and response variable. In addition, a comparison was made between the experimental values and predict data to determine the adequacy of the response regression equations as indicated by Montgomery [22].

## 4. Conclusion

The main and interaction effects of independent variables which are important in serine protease purification were investigated on response variables. RSM is demonstrated to be a useful method of optimization for purification of serine protease using PEG/dextran ATPS. The results show that PEG molecular weight has the most significant (*p* < 0.05) effect on partitioning behavior of serine protease, so PEG molecular weight should be considered as an important parameter for purification of the enzyme. Also, the addition of NaCl significantly affects enzyme partitioning to the top phase. The optimum condition for purification of serine protease was obtained at 8000 g·mol^−1^ PEG molecular weight, 17.2% (w·w^−1^) TLL, 4.5% (w·w^−1^) NaCl and pH 7.5. This system also provided the highest partition coefficient (84.2), purification factor (14.37) and yield (97.3) under this condition. Therefore, this study indicates that the partition coefficient, purification factor and the protease yield can be controlled by changing the main operating factors in the PEG/dextran ATPS system.

## Figures and Tables

**Table 1:** *F*-ratio and *p*-value for each independent variable effect in the polynomial response surface models.

Variables		Main effects				Quadratic effects				Interaction effects					
		*x*_1_	*x*_2_	*x*_3_	*x*_4_	x_1_^2^	*x*_2_^2^	*x*_3_^2^	*x*_4_^2^	*x*_1_*x*_2_	*x*_1_*x*_3_	*x*_1_*x*_4_	*x*_2_*x*_3_	*x*_2_*x*_4_	*x*_3_*x*_4_
Partition	*p*-value	0.000 a	0.001 a	_	_	_	0.002 a	_	_	0.001^a^	_	_	_	_	_
coefficient (*Y*_1_)	*F*-ratio	404.01	225.00	_	_	_	151.29	_	_	104.04	_	_	_	_	_
Purification	*p*-value	_	_	0.003 a	0.001 a	_	_	0.010 a	0.002 a	_	_	_	_	0.000 a	_
factor (*Y*_2_)	*F*-ratio	_	_	100.80	129.96	_	_	25.50	53.39	_	_	_	_	88.36	_
Yield	*p*-value	_	0.000 a	_	0.002 a	_	0.001 a	_	0.000 a	0.000 a	0.020 a	0.023 a	_	_	_
(*Y*_3_, %)	*F*-ratio	_	146.06	_	178.22	_	310.81	_	79.03	395.61	17.64	12.78	_	_	_

*x*_1_, *x*_2_, *x*_3_ and *x*_4_: Main effect of PEG molecular mass, TLL, NaCl and pH, respectively. *x*_1 2_, *x*_2 2_, *x*_3 2_ and *x*_4 2_: Quadratic effect of PEG molecular mass, TLL, NaCl and pH, respectively. *x*_1_*x*_2_: Interaction effect of PEG molecular mass and TLL. *x*_1_*x*_3_: Interaction effect of PEG molecular mass and NaCl. *x*_1_*x*_4_: Interaction effect of PEG molecular mass and pH. *x*_2_*x*_3_: The interaction effect of TLL and NaCl. *x*_2_*x*_4_: Interaction effect of TLL and pH. *x*_3_*x*_4_: Interaction effect of NaCl and pH.

a Significant (*p* < 0.05).

**Table 2:** Regression coefficient, *R*^2^, adjusted *R*^2^, probability values of the response surface models.

Regression coefficient	Partition coefficient (*Y*_1_)	Purification factor (*Y*_2_)	Yield (*Y*_3_ %)
*β*_0_	23.33	69.42	97.04
*β*_1_	4.50	_	_
*β*_2_	3.00	_	33.55
*β*_3_	_	4.22	_
*β*_4_	_	5.75	14.55
*β*_12_	_	_	_
*β*_22_	5.61	3.85	46.71
*β*_32_	_	_	_
*β*_42_	_	1.78	3.76
*β*_12_	1.62	_	18.50
*β*_13_	_	_	12.34
*β*_14_	_	_	14.80
*β*_23_	_	_	_
*β*_24_	_	8.82	_
*β*_34_	_	_	_
*R*^2^	0.997	0.993	0.996
*R*^2^ (adj.)	0.993	0.991	0.992
Regression(*p*-value)	0.001 a	0.000 a	0.000 a

*β_i_*: The estimated regression coefficient for the main linear effects. *β_ii_*: The estimated regression coefficient for quadratic effects. *β_ij_*: The estimated regression coefficient for the interaction effects. 1: PEG molecular mass; 2: TLL; 3: NaCl; 4: pH.

a Significant (*p* < 0.05).

**Table 3:** Matrix of the central composite design (CCD).

Treatment runs	Blocks	PEG molecular mass (g·mol^−1^)	TLL [% (w w^−1^)]	NaCl [% (w·w^−1^)]	pH
1	1	8000	17.20	11.5	7.5
2	1	8000	17.20	−2.5	7.5
3	1	8000	17.20	4.5	4.5
4	1	8000	−3.42	4.5	7.5
5	1	8000	35.27	4.5	7.5
6 c	1	8000	17.20	4.5	7.5
7	1	12,000	17.20	4.5	7.5
8	1	8000	17.20	4.5	7.5
9	1	8000	17.20	4.5	10.5
10 c	1	4000	17.20	4.5	7.5
11	2	10,000	6.25	1.0	6.0
12	2	6000	6.25	8.0	6.0
13	2	10,000	25.60	8.0	6.0
14	2	6000	25.60	1.0	6.0
15 c	2	10,000	25.60	1.0	9.0
16	2	8000	17.20	4.5	7.5
17	2	8000	17.20	4.5	7.5
18	2	6000	6.25	1.0	9.0
19	2	6000	25.60	8.0	9.0
20 c	2	10,000	6.25	8.0	9.0
21	3	8000	17.20	4.5	7.5
22	3	10,000	25.60	1.0	6.0
23	3	10,000	6.25	1.0	9.0
24	3	6000	6.25	8.0	9.0
25	3	6000	6.25	1.0	6.0
26	3	8000	17.20	4.5	7.5
27	3	10,000	25.60	8.0	9.0
28 c	3	6000	25.60	1.0	9.0
29	3	10,000	6.25	8.0	6.0
30 c	3	6000	25.6	8.0	6.0

c center poin.
